# Reinforcement of PMMA Denture Base Material with a Mixture of ZrO_2_ Nanoparticles and Glass Fibers

**DOI:** 10.1155/2019/2489393

**Published:** 2019-01-28

**Authors:** Mohammed M. Gad, Ahmad M. Al-Thobity, Ahmed Rahoma, Reem Abualsaud, Fahad A. Al-Harbi, Sultan Akhtar

**Affiliations:** ^1^Lecturer, Department of Substitutive Dental Sciences, College of Dentistry, Imam Abdulrahman Bin Faisal University, P.O. Box 1982, Dammam 31411, Saudi Arabia; ^2^Assistant Professor, Department of Substitutive Dental Sciences, College of Dentistry, Imam Abdulrahman Bin Faisal University, P.O. Box 1982, Dammam 31411, Saudi Arabia; ^3^Assistant Professor, Department of Restorative Dental Sciences, College of Dentistry, Imam Abdulrahman Bin Faisal University, P.O. Box 1982, Dammam 31411, Saudi Arabia; ^4^Professor, Department of Substitutive Dental Sciences, College of Dentistry, Imam Abdulrahman Bin Faisal University, P.O. Box 1982, Dammam 31411, Saudi Arabia; ^5^Department of Biophysics, Institute for Research and Medical Consultations, Imam Abdulrahman Bin Faisal University, P.O. Box 1982, Dammam 31411, Saudi Arabia

## Abstract

This study is aimed at evaluating the hybrid reinforcement effects of zirconium oxide nanoparticles (nano-ZrO_2_) and glass fibers (GFs) at different ratios on the flexural and impact strengths of a polymethylmethacrylate (PMMA) denture base. A total of 160 specimens were fabricated from heat-polymerized acrylic resins using the water bath technique. For the control group, the specimens did not receive any additions; for the test group, different concentrations of nano-ZrO_2_/GFs at 5% of the PMMA polymer were added. The concentrations of nano-ZrO_2_/GFs were as follows: 5%–0%, 4%–1%, 3%–2%, 2.5%–2.5%, 2%–3%, 1%–4%, and 0%–5%. The flexural strength was measured using the three-point bending test. The impact strength was measured using the Charpy impact test. Results were tabulated and analyzed using one-way analysis of variance (ANOVA) and the Tukey–Kramer multiple comparison test (*p* ≤ 0.05). The flexural and impact strengths of PMMA-nano-ZrO_2_ + GF composites were significantly improved when compared with those of pure PMMA (*p* < 0.05). The maximum flexural strength (94.05 ± 6.95 MPa) and impact strength (3.89 ± 0.46 kJ/m^2^) were obtained with PMMA (2.5%)/nano-ZrO_2_ + 2.5% GF mixtures and could be used for removable prosthesis fabrication.

## 1. Introduction

Polymethylmethacrylate (PMMA) resin is the most extensively used material for the fabrication of dentures because it possesses a combination of favorable characteristics, such as the ease of laboratory manipulation, light weight, inexpensive fabrication, stability in the oral environment, appropriate esthetic and color-matching ability, and lack of toxicity [1, 2]. However, it may not be ideal in every aspect and has several drawbacks that need to be addressed due to its poor mechanical performance. These include low impact resistance and fatigue failure. Acrylic dentures frequently fracture during service due to their poor strength characteristics [2, 3]. Structural modifications to the composition, such as the addition of fillers, could enhance the resin's mechanical properties [3, 4].

These modifications include the addition of different reinforcing fibers to improve the flexural and impact strengths of the composite resin material as well as its resistance to fatigue, which may extend the functional life of the acrylic prosthesis [5]. Previous studies showed that glass fibers (GFs) were superior to other fiber types (such as nylon, polyethylene, and aramid carbon/graphite fibers) that may have poor esthetics, exhibit weak adhesion with the resin matrix, or be impractical for dental laboratory practice [5–7]. On the other hand, GFs are known for their biocompatibility, acceptable appearance, and excellent mechanical properties [7, 8]. It has been reported that the mechanical properties of an acrylic composite depend on the bond between the resin matrix and the reinforcing GFs [8]. To attain a high level of adhesion between the two materials, the surface treatment of GFs with a silane coupling agent before incorporation into the resin matrix may result in positive findings [8, 9]. Many studies reported improvements in the flexural and impact strengths of PMMA/silane-treated GFs compared to those of similar composites with untreated GFs [9–12].

Recently, there has been an increasing trend toward incorporating ceramic fillers into denture-base acrylic resins to act as the reinforcing material. The aim of this addition is to achieve a composite resin material with more favorable mechanical properties [13]. Recently, zirconium oxide nanoparticles (nano-ZrO_2_) received attention because of their excellent biocompatibility as well as their white color, which makes them less likely to alter the esthetics in comparison to other metal oxide nanoparticles [13, 14]. The selection of nano-ZrO_2_ as a filler in this study was based on their ability to improve the mechanical properties of acrylic resins [13]. ZrO_2_ particles possess a variety of beneficial properties, such as excellent toughness and strength, abrasion and corrosion resistance, and biocompatibility [14, 15]. ZrO_2_ particles have a crystalline structure and have been reported for having high mechanical properties; being the hardest among any other oxides, they are able to withstand crack propagation [16, 17]. There are many factors that affect the mechanical and physical properties of the PMMA/nano-ZrO_2_ composite, such as shape, size, proportion, distribution, and composition of the matrix [14, 18]. Previous studies showed the effects of ZrO_2_ fillers on the properties of PMMA denture base material and found that nano-ZrO_2_ have the ability to significantly increase the flexural and impact strengths of the acrylic denture base [14, 19]. The greatest increase was observed in a denture base nanocomposite containing 5 wt.% of nano-ZrO_2_ [20]. The increase in the nanofiller concentration beyond 5 wt.% resulted in particle agglomerations and cluster formations that weakened the material rather than strengthening it [13].

Hybrid reinforcement systems have been created previously [13, 21] to develop mixtures of different fibers, metal oxides, or fibers and fillers reported to produce improvements in the physical properties compared to adding them separately [13, 21]. Hybrid reinforcement can be generated by one of the following methods: adding a mixture of more than one type of fiber [22], combining a variety of metal oxides and ceramics [23, 24], adding mixtures of metal oxides and fibers [25, 26], or using a combination of ceramic fillers [21, 27, 28]. A previous study reported promising results for the flexural strength and toughness of acrylic resins reinforced with a hybrid of fiber-reinforcing materials [16]. The addition of hybrid reinforcement of fibers and fillers was found to improve the impact strength as well [25]. Although the incorporation of nano-ZrO_2_ and GFs into PMMA to improve its physical and mechanical properties has been done separately, to the knowledge of the authors, no studies have yet been conducted to evaluate the effect of nano-ZrO_2_/GFs mixture's reinforcement on the mechanical properties of heat-cured acrylic denture base resins. Therefore, this study was conducted to evaluate the flexural and impact strengths of acrylic denture base resins reinforced with a mixture of nano-ZrO_2_ and GFs at varying concentrations. The null hypothesis in this study is that the mixture of nano-ZrO_2_ and GFs would not improve the mechanical properties of the acrylic denture base resin.

## 2. Materials and Methods

### 2.1. Specimen Preparation

Two different metal molds were constructed in the desired shape and dimensions for each test. The molds were used to create wax-up specimens (Cavex Set Up Wax, Cavex), and 160 specimens were created (eighty specimens per test). Wax specimens were deposited in a dental stone (Fujirock EP, GC) within a bottom flask (61B Two Flask Compress, Handler Manufacturing). A petroleum jelly separating medium was applied to the stone's surface before positioning the top flask and filling it with another layer of stone. After the stone set, the flasks were placed in a wax elimination machine for 5 minutes. The separated halves of the flask were then cleaned under running hot water to remove wax traces and create mold spaces. While the stone surface was still hot, a separating medium (Isolmajor, Major Prodotti Dentari SPA) was applied, and the stone surface was set aside for packing.

### 2.2. GF Specifications and Treatment

**Figure 1:**
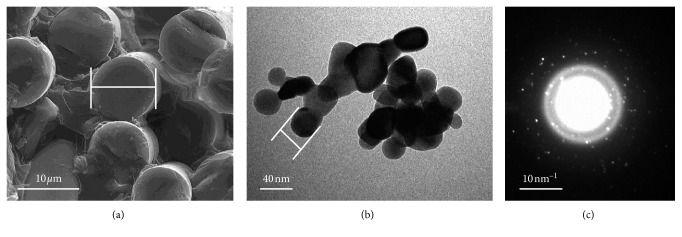
(a) SEM showing diameter and size analysis of glass fibers (GFs) (∼12 *µ*m); (b) TEM showing the size of zirconium oxide nanoparticles (nano-ZrO_2_) (∼40 nm); and (c) spots in the TEM electron diffraction pattern of ZrO_2_ nanoparticles showing the crystalline nature of the particles.

GFs (E-glass, Shanghai Richem International Co., Ltd.) 3 mm in length and 12 *μ*m in diameter (Figure 1) were weighed using an electronic balance (S-234, Denver Instrument) to create different concentrations of acrylic powder/GF mixtures (Table 1). Preweighed GFs were soaked in a silane coupling agent (3-trimethoxysilyl propyl methacrylate, 97% (TMSPM), Shanghai Richem International Co., Ltd.) for 1 min at room temperature and were then dried at 60°C for 24 h [29].

### 2.3. Nano-ZrO_2_ Specifications and Treatment

Nano-ZrO_2_ (99.9%, 100 nm, 1314-23-4, Shanghai Richem International Co., Ltd.) with a surface area of 9 ± 2 m^2^/g and an average size of 40 ± 3 nm (Figure 1) were treated with 0.3 g of TMSPM. This process allowed for adequate adhesion between the resin matrix and nano-ZrO_2_ [14].

### 2.4. Mixture Preparation

The amount of nano-ZrO_2_ + GFs addition was fixed at 5 wt.% of the acrylic powder (Major Base 20, Major Prodotti Dentari SPA). However, the ratio of nano-ZrO_2_ + GFs added is described in Table 1. The sum of preweighed treated nano-ZrO_2_ and GFs percentages allocated per group was added to heat-cured acrylic resin powder in a plastic beaker, forming 100% of the mixture for each group (5% of acrylic powder). The mixtures were stirred with a blender at a speed of 400 rpm for 30 min to achieve an even distribution of nano-ZrO_2_ and GFs within the acrylic powder and obtain a consistent color.

### 2.5. Specimen Processing

According to the manufacturer's instructions, a polymer/monomer ratio of 3 : 1 by volume was combined, mixed, and set aside until it reached a dough state and then packed in mold spaces and pressed for 30 minutes in a hydraulic press at 30 MPa. For polymerization, flasks were placed into a thermostatically controlled water bath (KaVo Elektrotechnisches Werk GmbH). Starting with cold water, the temperature was increased to 70°C for 90 minutes followed by 100°C for 30 minutes and then allowed to cool to room temperature for 1 h. After cooling, the specimens were retrieved, finished with a thin cross-cut tungsten carbide bur (HM251 FX 040 HP) at 18,000 rpm, and polished with a coarse grain cylindrical rubber tip bur for acrylic resin polishing (Super Acrylic Polish, Long Dental) followed by a fine grain cylindrical rubber tip bur (Super Acrylic Polish, Long Dental). A soft bristle brush with fine pumice dust (Steribim Super, Bego, Wilhim-Herbst-strabe 1) mixed with an equal volume of water was used for final polishing. A digital caliper with an accuracy of 0.01 mm (extra large LCD screen digital caliper, Neiko tool) was used to evaluate the dimensions of the prepared specimens. The approved 160 specimens were divided into eight groups per test. Each test group had 10 specimens (*n* = 10) (Table 1). The finished and polished specimens were stored in distilled water at 37°C for 48 h prior to testing.

### 2.6. Flexural Strength Test

**Figure 2:**
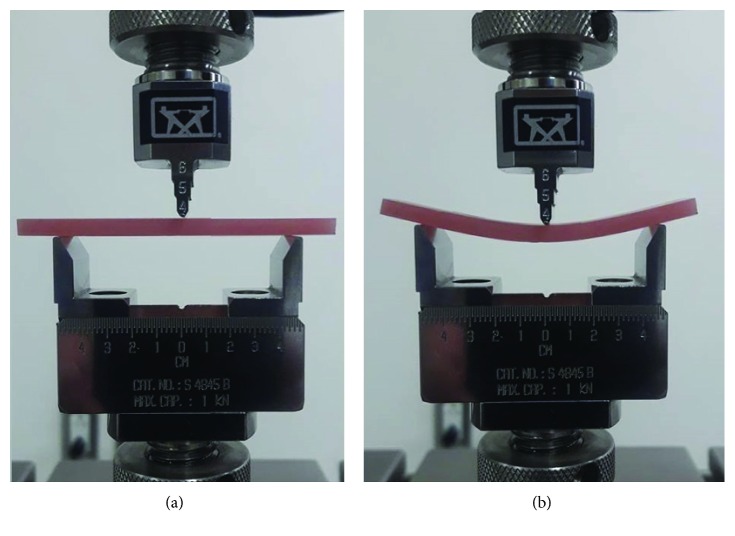
(a) Flexural strength of the specimen placed on the universal testing machine and (b) specimen subjected to bending strength until failure load recorded with specimen fracture.

For the flexural strength test, the specimens were bar-shaped with dimensions of 65 × 10 × 2.5 mm ± 0.2 mm according to American Dental Association (ADA) Specification No. 12 [30]. The specimens were retrieved from the water and they underwent the three-point bending test while still wet using a universal testing machine (Instron 8871, Instron Co.). Each specimen was rested on two support pins with 50 mm spans. A 490 newton load cell was used to apply force at the center of the opposing surface at a crosshead speed of 5 mm/min (Figures 2(a) and 2(b)). The load at fracture was recorded, and the flexural strength of the specimen was calculated using the equation:

(1) $$\begin{array}{c} S=\frac{3WL}{2bd^{2}}, \end{array}$$

where *S* is the value of the flexural strength measured in MPa, *W* is the load at fracture in newton, *L* is the support separation distance (50 mm), *b* is the width of the specimen (10 mm), and *d* is the thickness (2.5 mm).

### 2.7. Impact Strength Test

**Figure 3:**
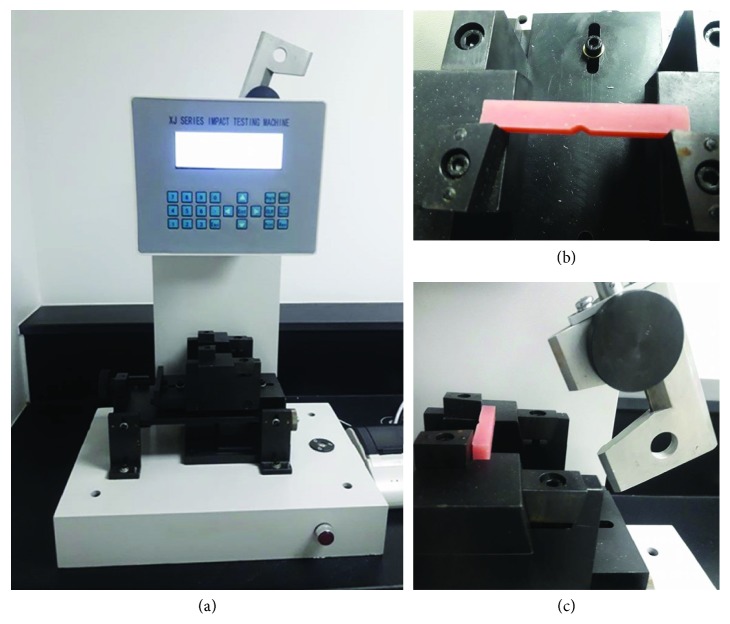
(a) Charpy's impact strength machine; (b) specimen placed horizontally where the un-notched side faces the hummer; (c) load released in a pendulum action to fracture the specimen and impact strength digitally recorded.

Specimens for this test were made according to ISO #1567 into bar-shaped blocks with dimensions of 55 × 10 × 10 mm with a V-shaped notch. The notch was 2.5 mm deep across the entire 10 mm width of the specimen, leaving an effective depth of 7.5 mm below the notch [31]. A pendulum Charpy-type impact test machine (Digital Charpy Izod impact tester, XJU 5.5, Jinan Hensgrand Instrument Co., Ltd.) was used to perform the impact test at room temperature. The specimen was secured in place horizontally using two support arms 40 mm away from each other (Figures 3(a)–3(c)). A 0.5 J drop weight was released at the midpoint on the opposite side of the notch, and the impact strength was recorded for each specimen in kJ/m^2^ [18, 20].

### 2.8. Scanning Electron Microscopy (SEM)

After the flexural and impact tests, the surface morphology of each cross section was examined using a scanning electron microscope (FEI, INSPECT S50). The scanning electron microscope was operated at 20 kV. The samples were gold-coated using a coating machine (Quorum, Q150R ES) to acquire high-quality electronic images. To obtain the illustrative information of each specimen, the images were taken at various magnifications: ×500, ×1000, ×2000, ×4000, and ×5000. The morphological features and structure of the reinforced agents, GFs, and nano-ZrO_2_ were also analyzed before their inclusion to the PMMA matrix (Figure 1). The glass fibers were examined by SEM to estimate the diameter of the individual fibers (12 *μ*m). The nano-ZrO_2_ particles were observed by transmission electron microscopy (TEM) (FEI, Morgagni 268). The transmission electron microscope was operated at 80 kV and recorded several images. More than 20 particles were measured to obtain the average size (40 ± 3 nm). Electron diffraction patterns of the nano-ZrO_2_ particles were also observed in the transmission electron microscope to confirm the crystalline nature of the particles.

The software package SPSS-20.0 (IBM, Armonk, NY) was used to perform statistical data analysis. The results of the flexural and impact strength tests were transformed into arithmetic means and standard deviation (SD). One-way ANOVA was performed to compare the flexural and impact strengths of the control and treatment groups, and a Tukey–Kramer multiple comparison test was performed for all pairwise differences between the means. A *p* value ≤0.05 was considered a statistically significant result.

## 3. Results

Mean values, standard deviations, and statistically significant differences of flexural and impact strengths are summarized in Table 2. The addition of different concentrations of nano-ZrO_2_ + GFs significantly increased the flexural strength for all reinforced groups when compared to the control group (*p* < 0.05). Different ratios of nano-ZrO_2_ + GFs resulted in varied effects on the flexural strength of the composite material. The flexural strength increased gradually from group A (5% nano-ZrO_2_ + 0% GFs) to group D (2.5% nano-ZrO_2_ + 2.5% GFs), and then a gradual decrease occurred, as shown from group E (2% nano-ZrO_2_ + 3% GFs) to group G (0% nano-ZrO_2_ + 5% GFs). Of the reinforced groups, group G (68.21 ± 7.76 MPa) showed the lowest mean value, followed by groups A (75.16 ± 6.95 MPa), F (75.55 ± 6.23 MPa), and B (77.63 ± 5.65 MPa), with no significant differences between these three groups. Additionally, groups E (83.28 ± 5.32 MPa) and C (85.82 ± 6.96 MPa) showed significantly high values for the flexural strength compared to groups A, F, and B. Finally, the highest flexural strength was seen in group D (94.05 ± 6.95 MPa).

As seen in Table 2, a significant increase in the impact strength of all reinforced groups was noted when compared to that of the control group (*p* < 0.05), except for group G (*p* > 0.05). The added nano-ZrO_2_ + GF mixture improved the impact strength, as seen in groups A, B, C, and D, where groups C and D had the highest significant values compared to the test group. On the other hand, a decrease in impact strength was recorded for groups E, F, and G. Group G (2.37 ± 0.46 kJ/m^2^) had the lowest impact strength value, which was not significantly different from that of the control group (1.99 ± 0.63 kJ/m^2^), with a *p* value >0.05.

### 3.1. SEM Analysis

**Figure 4:**
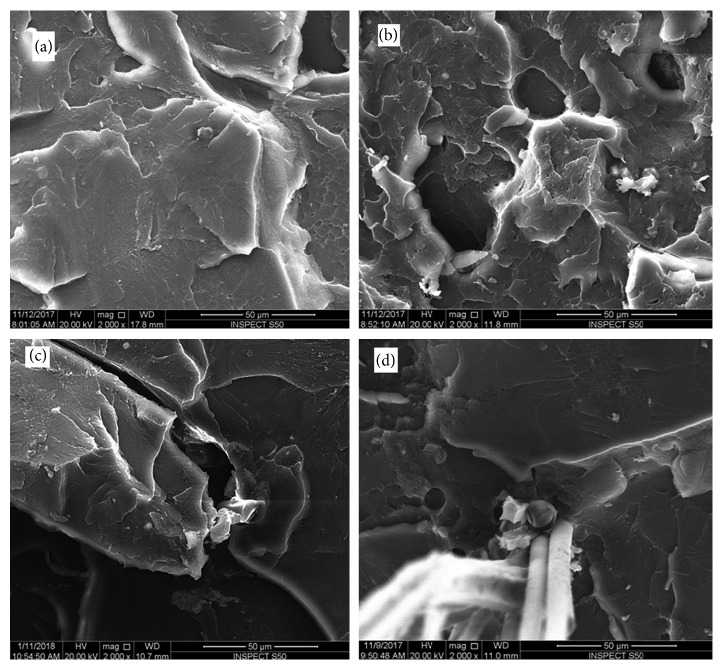
SEM of fracture surfaces of flexural test specimens. (a) Control-0% nano-ZrO_2_ + 0% GFs; (b) 5% nano-ZrO_2_ + 0% GFs; (c) 4% nano-ZrO_2_ + 1% GFs; (d) 3% nano-ZrO_2_ + 2% GFs.

**Figure 5:**
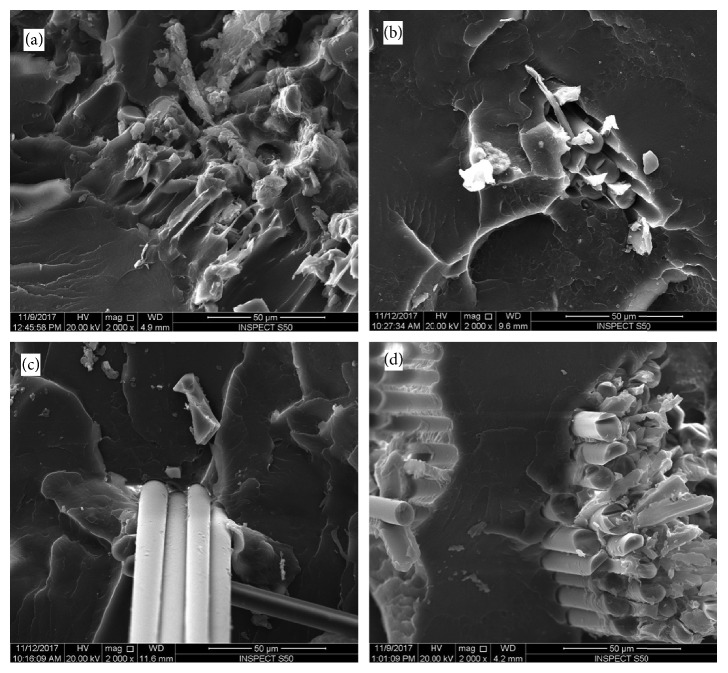
SEM of fracture surfaces of flexural test specimens. (a) 2.5% nano-ZrO_2_ + 2.5% GFs; (b) 2% nano-ZrO_2_ + 3% GFs; (c) 1% nano-ZrO_2_ + 4% GFs; (d) 0% nano-ZrO_2_ + 5% GFs.

**Figure 6:**
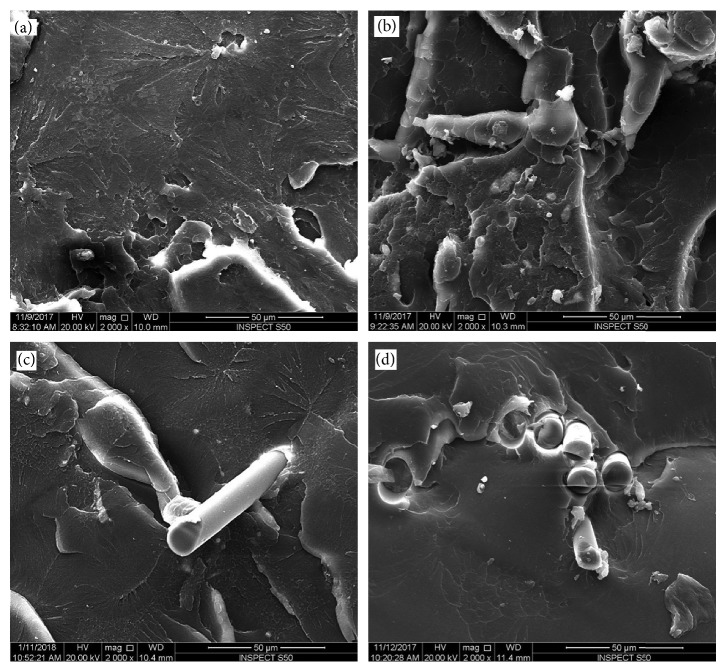
SEM of fracture surfaces of impact test specimens. (a) Control-0% nano-ZrO_2_ + 0% GFs; (b) 5% nano-ZrO_2_ + 0% GFs; (c) 4% nano-ZrO_2_ + 1% GFs; (d) 3% nano-ZrO_2_ + 2% GFs.

**Figure 7:**
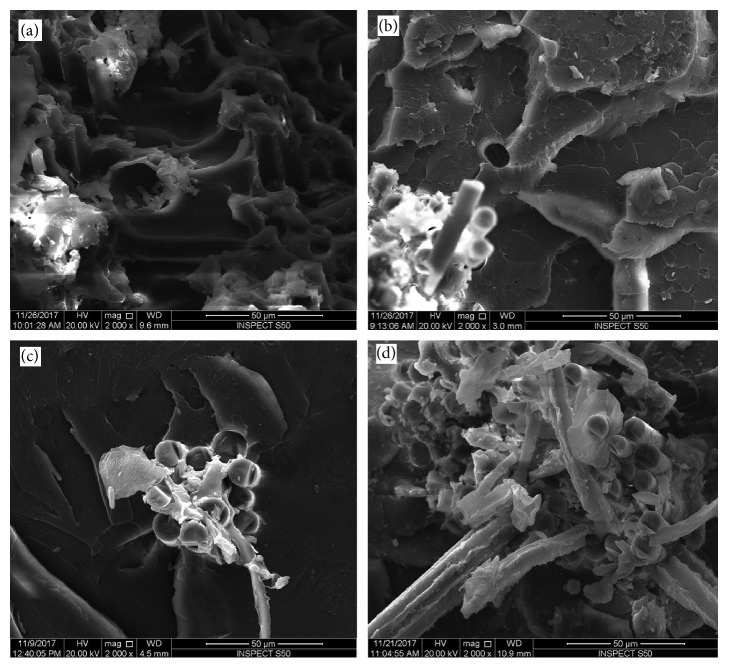
SEM of fracture surfaces of impact test specimens. (a) 2.5% nano-ZrO_2_ + 2.5% GFs; (b) 2% nano-ZrO_2_ + 3% GFs; (c) 1% nano-ZrO_2_ + 4% GFs; (d) 0% nano-ZrO_2_ + 5% GFs.

The surface of each fractured sample was assessed using SEM (Figures 4–7). SEM assessment was performed on the fractured end of the representative samples from each group, and the following observations were revealed to correlate with mechanical properties of the matrix. SEM micrograph of the control specimen (no addition of reinforced agents) exhibited a flake-like morphology and a smooth surface (Figure 4(a)) compared to other tested specimens. This is an indication of a brittle mode of fracture. Upon addition of nano-ZrO_2_ and GFs to the PMMA (groups A–G), the samples showed a comparatively rough surface with varying morphological features, indicating both ductile and brittle modes of failure. SEM view of the group D specimens (Figure 5(a)) showed the roughest surface among all the reinforced groups. The glass fibers failed adhesively at the plane of the fracture and protruded. The voids formed due to the glass fibers that were pulled out were higher in number than group C. The SEM image of group E specimens exhibited a similar morphology to that seen with group C, but the fibers were completely protruding out of the fractured end with voids on the opposing surface of the specimens (Figure 5(b)). As expected, a considerable number of GFs were seen on the surface of the specimens of groups F and G (Figures 5(c) and 5(d)).

Some variations in the surface morphological features were noticed when comparing SEM micrographs of specimens undergoing flexural tests and those undergoing impact tests (Figures 6 and 7). The specimens of groups B and C (Figures 6(c) and 6(d)) showed rough surfaces compared to the control group (Figure 6(a)), with few fractured GFs. However, the number of GFs was higher for group C specimens. The surfaces of group D and G specimens displayed comparable morphology with the roughest surfaces among all the groups (Figures 7(a) and 7(d)). However, group G specimens showed a higher density of GFs than group D. No obvious surface cracks were observed for these specimens. In addition, a similarity in the surface morphology was noticed between the specimens of groups E and F (Figures 7(b) and 7(c)). Both surfaces showed a bundle of fractured GFs.

**Figure 8:**
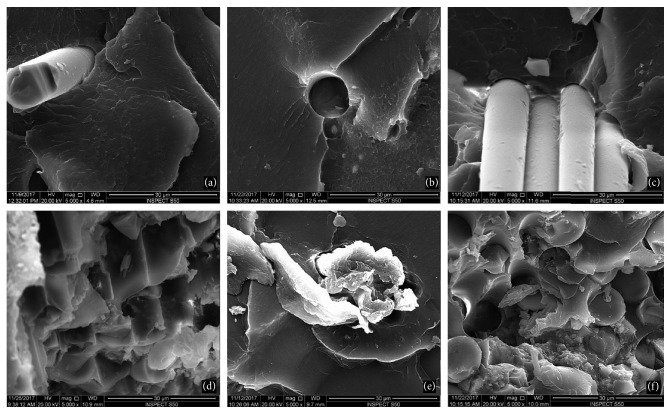
(a–f) SEM of fracture surfaces of PMMA-nano-ZrO_2_ + GF nanocomposites for some specimens with high magnification of 5000x. (a), (c), and (e) show some fibers protruding out of the fracture surfaces while (d) and (f) show the voids created by pulled-out fiber from the other side of the fracture surface.

Fiber detachment during fracture presented an adhesive type with hollow spaces between GFs and the resin matrix (Figures 8(a), 8(c), and 8(e)) in addition to fiber pull out leaving void spaces on the other side (Figures 8(b) and 8(d)), with cohesive fracture of GFs at the fracture site (Figure 8(f)). Also, there was noticeable nano-ZrO_2_ distribution around the fibers or fiber spaces (Figures 8(d) and 8(f)).

## 4. Discussion

Various kinds of polymers reinforced with nanoparticles and fibers have a wide range of applications [13]. Polymer composites reinforced with different types of nanoparticles or different types of fibers have been investigated with the expectation that the PMMA/composite material may lead to revolutionary means of achieving properties that cannot be provided with one reinforcement type [26, 32]. The nanocomposite materials have many favorable mechanical properties, making them suitable for various industrial uses such as optics, electronics, ionics, mechanics, membranes, functional and protective coatings, catalysis, sensors, biology, medicine, and biotechnology [13, 27]. This encourages scientists to explore the effects, such as flexural strength and impact strength, of different types of reinforcing materials on the mechanical properties of nanocomposite materials [21]. Due to the advantages of nanomaterials, several studies showed interest in utilizing them to improve the mechanical properties of PMMA [33, 34]. Additionally, glass fibers possess high mechanical properties and are biologically acceptable. Moreover, their addition to the PMMA denture base material improves its mechanical properties [13, 29]. Therefore, the current study selected nano-ZrO_2_ and GFs and studied the effects of different ratios of hybrid reinforcement on flexural and impact strengths. In the current study, surface treatment of the nano-ZrO_2_ and GFs with the bifunctional silane coupling agent TMSPM was performed. This agent has functional hydroxyl groups that bond to the fillers and fibers in addition to the presence of C=C bonds, which react with PMMA during polymerization and bond them to nano-ZrO_2_ and GFs [26, 28]. Results showed that the PMMA composites with nano-ZrO_2_ and GFs improved the mechanical properties of the PMMA denture base material; therefore, the null hypothesis was rejected.

The results showed that the flexural strength of group A (5% nano-ZrO_2_ + 0% GFs) nanocomposites was elevated by 21% when compared to that of unreinforced PMMA. The good distribution of the very finely sized nano-ZrO_2_ used in the study enabled them to occupy the spaces between linear chains of the polymer, thereby restricting the segmental motions of the macromolecular chains and increasing strength and rigidity of the resin. This mechanism enhanced the fracture resistance and improved flexural strength [16, 17]. In addition, the increase in flexural strength could be due to transformation toughening. When sufficient stress develops and a microcrack begins to propagate, a transformation of nanoparticles from the tetragonal crystalline phase to the monoclinic phase occurs, depleting the energy of the crack and stopping its propagation. In this process, expansion of crystals occurs, placing the crack under a state of compression and arresting its propagation [16]. This finding conforms to that obtained by Safarabadi et al. [23], Alhareb and Ahmed [27], and Zhang et al. [35]. Zhang et al. [35] studied the effects of nano-ZrO_2_ on the flexural strength of PMMA and found that PMMA/nano-ZrO_2_ composites reached the highest flexural strength when nano-ZrO_2_ was added at 1.5 wt.%, with a 23% increase in flexural strength compared with pure PMMA.

The flexural strength of group G (0% nano-ZrO_2_ + 5% GFs) nanocomposites was elevated by 6% when compared to that of pure PMMA. When the load is applied on the specimen, tension occurs below the long axis of the specimen. The high modulus of elasticity of GFs as well as the strong bond between the matrix and fibers leads to hindrance of crack initiation and propagation under the failure load; resist tension occurs below the long axis of the specimens, subsequently increasing the flexural strength. Moreover, the chemical bond between GFs and the resin matrix stops the elongation of the polymer matrix [36]. The results of the current study agree with those of a previous study by Yu et al. [22], which reported that the addition of GFs increased the flexural strength of the PMMA denture base material.

Adding the nano-ZrO_2_/GF mixture in different concentrations increased the flexural strength. The flexural strength of group D (2.5% nano-ZrO_2_ + 2.5% GFs) nanocomposites was increased by 45% when compared to that of unreinforced PMMA. Therefore, the reinforcing effect of the nano-ZrO_2_/GFs composite was more effective. This was possible because of the synergistic effect of nano-ZrO_2_ and GFs in enhancing the mechanical properties of PMMA. As presented in Table 2, the addition of nano-ZrO_2_/GFs to PMMA showed a significant increase in flexural strength as compared to that of the control group. The maximum flexural strength value was seen in group D (2.5% nano-ZrO_2_ + 2.5% GFs). Changing the mixture ratio resulted in variation of flexural strength values. As the nano-ZrO_2_ or GFs addition increased (away from 2.5% nano-ZrO_2_ + 2.5% GFs), a continuous decrease in the flexural strength was observed for groups B, C, E, and F.

When the amount of GFs increased more than 2.5%, the flexural strength decreased. This started to show in group E (2% nano-ZrO_2_ + 3% GFs), followed by group F (1% nano-ZrO_2_ + 4% GFs), and finally group G (0% nano-ZrO_2_ + 5% GFs), which showed the lowest flexural strength value. The effect of nano-ZrO_2_ decreased as their amount decreased. In addition, bundle formation of GFs started to occur as their concentration increased. It was found that an inverse relationship exists between the content of nano-ZrO_2_/GFs and the flexural strength away from the 2.5% nano-ZrO_2_ + 2.5% GFs percentage. Higher amounts of GFs resulted in agglomeration in bundles, which eventually weakened the material (Figures 5(c), 5(d), 7(c), and 7(d)). In the same manner, increasing nano-ZrO_2_ and decreasing GFs caused the GFs to lose their positive effect in the composite. However, even with the decrease in the flexural strength of group A (5% nano-ZrO_2_ + 0% GFs) and group G (0% nano-ZrO_2_ + 5% GFs) compared to that of group D (2.5% nano-ZrO_2_ + 2.5% GFs), flexural strength results were still significantly higher than the control group, confirming the effect of reinforcement with nano-ZrO_2_ or GFs in agreement with previous studies [7, 8, 11, 35, 37].

The results of the present study confirmed that at certain percentages of 2.5% nano-ZrO_2_ + 2.5% GFs, the flexural strength was at its maximum. The increase in flexural strength with these percentages could be attributed to the synergistic effect of nano-ZrO_2_ and GFs. Altering the ratio (increasing or decreasing) from this optimal level led to a decrease in the flexural strength. This is confirmed using the SEM investigation of the fractured surfaces. Figures 4(d), 5(a), 5(b), 6(c), 7(b), and 7(c) confirm the ductile fracture of the specimens with the presence of lamellae and fair distribution of nano-ZrO_2_ particles. A homogenous distribution of nano-ZrO_2_ particles within the resin matrix (Figures 4(b), 4(c), 6(b), and 6(c)) proved their positive effect in improving the flexural strength of the nanocomposite. Furthermore, GF fractures at the failure site revealed good adhesion between GFs and the resin matrix (Figures 5(d), 6(d), 7(b), and 7(c)). However, when the amount of GFs was at its maximum (0% nano-ZrO_2_ + 5% GFs), GFs collected as bundles, resulting in poor adhesion to the resin matrix and detaching easily from the resin matrix, leaving large voids (Figures 5(c), 5(d), 7(c), and 7(d)). At the end, the flexural strength deteriorated in comparison to that of other hybrid nanocomposite groups but was still higher than that of the control group.

The impact strength increased with nano-ZrO_2_ addition in comparison to the control group. This was due to the improvement of mechanical properties associated with the addition of ZrO_2_ nanoparticles, including mechanisms of crack refraction and crack restraining [14, 16, 38]. Hameed and Abdul Rahman [39] studied the effect of nano-ZrO_2_ addition to the PMMA denture base material and found a significant increase in impact strength with 5% nano-ZrO_2_, which the results of the current study agree with. As seen in group G, the addition of GFs caused a slight insignificant increase of impact strength in comparison to that of the control group, and this is in disagreement with previous studies [6, 7, 9, 12] that reported a significant increase in the impact strength with the addition of GFs to PMMA. This difference may be attributed to different types of fibers (woven type) used, the method of addition, or the denture base material used.

The addition of nano-ZrO_2_ + GFs to PMMA significantly increased the impact strength in comparison to the control group, except for group G (0% nano-ZrO_2_ + 5% GFs). The highest impact strength was observed in group D (2.5% nano-ZrO_2_ + 2.5% GFs). The impact strength of group D (2.5% nano-ZrO_2_ + 2.5% GFs) nanocomposites was increased by 51% when compared to that of unreinforced PMMA. It was noticed that the reinforcement procedure was affected by the size and surface properties of nano-ZrO_2_ and their ability to fill minute spaces in polymer chains and to undergo transformation toughening. In addition, the modulus of elasticity of GFs and their high mechanical properties had a major impact on reinforcement procedures. Furthermore, the surface modification of the nano-ZrO_2_ and GFs with a TMSPM coupling agent provided better distribution of particles in the matrix, preventing agglomeration and improving interfacial adhesion of the fillers to the polymer matrix. Moreover, the formation of cross links, or supramolecular bonds, prevented the propagation of cracks by transferring the stress from the matrix to the nanofillers and GFs [12, 37]. Additionally, the nanoparticles were characterized by a large specific surface area; thus, they had the ability to dissipate energy, which in turn may have enhanced the impact strength. The results of this study agree with those obtained by Safarabadi et al. [23].

In this study, the values of impact strength of the hybrid composite specimens gradually decreased with every increase in the volume fraction of nano-ZrO_2_/GFs moving from 2.5% nano-ZrO_2_ and 2.5% GFs (group D). Away from this percentage, the values of impact strength decreased for groups B, C, E, and F. When GFs or nano-ZrO_2_ increased more than 2.5%, the impact strength of groups B, E, F, and G significantly decreased in comparison to that of group D (2.5% nano-ZrO_2_ + 2.5% GFs), while group G (0% nano-ZrO_2_ + 5% GFs) showed the lowest impact strength value. This might be linked to the fact that high concentrations of GFs are susceptible to bundle formation within the PMMA matrix, lowering the impact strength of the hybrid reinforced specimens, particularly for group G (0% nano-ZrO_2_ + 5% GFs). These bundles may be seen as a separate bundle, agglomerate forming clusters within the PMMA, or superficially present and acting as stress concentration areas.

Clinically, a high mechanical performance is of prime importance. According to the results of this study, nano-ZrO_2_ and GFs could be added to denture base materials for enhancement. The optimum level of reinforcement that produced a composite denture base material with adequate mechanical properties for removable prosthesis fabrication was determined to be 2.5% nano-ZrO_2_ + 2.5% GFs. The meticulous incorporation of those specific amounts resulted in better flexural and impact strengths of the hybrid material. The limitations of this study include the following: fixed concentration of additions to the acrylic resin powder, one type of denture base material tested, only two mechanical tests conducted, and the testing conditions did not exactly mimic the oral environment. Therefore, further investigation with different concentrations, different types of denture base materials, and ageing procedures in conditions simulating the oral environment are recommended. Furthermore, the addition of this nanocomposite to acrylic teeth may improve its physical properties; therefore, we recommended further investigation on the additional effects and physical properties of the newly introduced nanocomposite mixture to acrylic teeth as well as acrylic removable appliances.

## 5. Conclusions

Within the limitations of this study, the following conclusions can be drawn:

1. The addition of nano-ZrO_2_, GFs, or nano-ZrO_2_ + GFs to PMMA denture base materials improves the flexural strength
2. The addition of nano-ZrO_2_ or nano-ZrO_2_ + GFs to PMMA denture base materials improves the impact strength
3. 95% PMMA + 2.5% nano-ZrO_2_ + 2.5% GF composites have the best balance of flexural strength and impact strength, and this ratio is recommended as the hybrid reinforcement for denture base materials

## Figures and Tables

**Table 1:** Specimen grouping according to filler, fiber, and acrylic powder percentages.

Group	Percentage of filler/fiber/acrylic powder	
Control	0% (0% nano-ZrO_2_ + 0% GFs)	100% acrylic powder
A	5% (5% nano-ZrO_2_ + 0% GFs)	95% acrylic powder
B	5% (4% nano-ZrO_2_ + 1% GFs)	95% acrylic powder
C	5% (3% nano-ZrO_2_ + 2% GFs)	95% acrylic powder
D	5% (2.5% nano-ZrO_2_ + 2.5% GFs)	95% acrylic powder
E	5% (2% nano-ZrO_2_ + 3% GFs)	95% acrylic powder
F	5% (1% nano-ZrO_2_ + 4% GFs)	95% acrylic powder
G	5% (0% nano-ZrO_2_ + 5% GFs)	95% acrylic powder

**Table 2:** Tukey–Kramer multiple-comparison test for flexural strength (MPa) and impact strength (kJ/m^2^) of denture base resins showing mean ± SD and groups with significant differences.

Groups	Flexural strength (MPa) Mean ± SD	Impact strength (kJ/m^2^) Mean ± SD
Control	64.52 ± 5.76	3.89 ± 0.46^a^
A	75.16 ± 6.95^a^	3.80 ± 0.71^b,c^
B	77.63 ± 5.65^a^	3.44 ± 0.82^b^
C	85.82 ± 6.96^b^	3.30 ± 0.65^c,d^
D	94.05 ± 6.95	3.24 ± 0.64^d^
E	83.28 ± 5.32^b^	2.77 ± 0.92^b^
F	75.55 ± 6.23^a^	2.37 ± 0.46^e^
G	68.21 ± 7.76	1.99 ± 0.63^a,e^

Multiple comparison tests for all pairwise differences between the means. Groups with similar letters are not significantly different from each other.

## Data Availability

The data used to support the findings of this study are available from the corresponding author upon request.

## References

[1] Carlsson G. E., Omar R. (2010). The future of complete dentures in oral rehabilitation: a critical review. *Journal of Oral Rehabilitation*.

[2] Meng T. R., Latta M. A. (2005). Physical properties of four acrylic denture base resins. *Journal of Contemporary Dental Practice*.

[3] Pratibha Y., Mittal R., Sood V. K. (2012). Effect of incorporation of silane-treated silver and aluminium micro particles on strength and thermal conductivity of PMMA. *Journal of Prosthodontics*.

[4] Mowade T. K., Dange S. P., Thakre M. B., Kamble V. D. (2012). Effect of fiber reinforcement on impact strength of heat polymerized polymethyl methacrylate denture base resin:in vitrostudy and SEM analysis. *Journal of Advanced Prosthodontics*.

[5] Alla R. K., Sajjan S., Alluri V. R., Ginjupalli K., Upadhya N. (2013). Influence of fiber reinforcement on the properties of denture base resins. *Journal of Biomaterials and Nanobiotechnology*.

[6] Gad M. M., Rahoma A., Al-Thobity A. M. (2018). Effect of polymerization technique and glass fiber addition on the surface roughness and hardness of PMMA denture base material. *Dental Materials Journal*.

[7] Uzun G., Hersek N., Tinçer T. (1999). Effect of five woven fiber reinforcements on the impact and transverse strength of a denture base resin. *Journal of Prosthetic Dentistry*.

[8] Goguta L. M., Bratu D., Topala F. (2006). Impact strength of acrylic heat curing denture base resin reinforced with E-glass fibers. *Temporomandibular Joint Disorders*.

[9] Vallittu P. K., Narva K. (1997). Impact strength of a modified continuous glass fiber-poly (methyl methacrylate). *International Journal of Prosthodontics*.

[10] Chen S.-Y., Liang W.-M., Yen P.-S. (2001). Reinforcement of acrylic denture base resin by incorporation of various fibers. *Journal of Biomedical Materials Research*.

[11] Vojdani M., Khaled A. R. (2006). Transverse strength of reinforced denture base resin with metal wire and E-glass fibers. *Journal of Dentistry*.

[12] Hari Prasad A., Kalavathy A., Mohammed H. S. (2011). Effect of glass fiber and silane treated glass fiber reinforcement on impact strength of maxillary complete denture. *Annals and Essences of Dentistry*.

[13] Gad M., Fouda S., Al-Harbi F., Näpänkangas R., Raustia A. (2017). PMMA denture base material enhancement: a review of fiber, filler, and nanofiller addition. *International Journal of Nanomedicine*.

[14] Ayad N. M., dawi M. F., Fatah A. A. (2008). Effect of reinforcement of high impact acrylic resin with micro-Zirconia on some physical and mechanical properties. *Revista de Clínica e Pesquisa Odontológica*.

[15] Gad M., ArRejaie A. S., Abdel-Halim M. S. (2016). The reinforcement effect of nano-zirconia on the transverse strength of repaired acrylic denture base. *International Journal of Dentistry*.

[16] Anusavice K. J. (2003). *Phillip’s Science of Dental Materials*.

[17] Gad M., Abualsaud R., Rahoma A., Al-Thobity A. M., Alabidi K., Akhtar S. (2018). Effect of zirconium oxide nanoparticles addition on the optical and tensile properties of polymethyl methacrylate denture base material. *International Journal of Nanomedicine*.

[18] Gad M., Rahoma A., Al-Thobity A. M., ArRejaie A. (2016). Influence of incorporation of ZrO_2_ nanoparticles on the repair strength of polymethyl methacrylate denture bases. *International Journal of Nanomedicine*.

[19] Neset V. A., Hamdi A., Turan K., Turkyilmaz I. (2013). Influence of various metal oxides on mechanical and physical properties of heat-cured polymethylmethacrylate denture base resins. *Journal of Advanced Prosthodontics*.

[20] Ihab N. S., Moudhaffar M. (2011). Evaluation the effect of modified nano-fillers addition on some properties of heat cured acrylic denture base material. *Journal of Baghdad College of Dentistry*.

[21] Salih S. I., Oleiwi J. K., Hamad Q. A. (2015). Investigation of fatigue and compression strength for the PMMA reinforced by different system for denture applications. *International Journal of Biomedical Materials Research*.

[22] Yu S.-H., Lee Y., Oh S., Cho H.-W., Oda Y., Bae J.-M. (2012). Reinforcing effects of different fibers on denture base resin based on the fiber type, concentration, and combination. *Dental Materials Journal*.

[23] Safarabadi M., Mehri Khansari N., Rezaei A. (2014). An experimental investigation of HA/AL2O3 nanoparticles on mechanical properties of restoration materials. *Engineering Solid Mechanics*.

[24] Basima M. A., Aljafery A. M. A. (2015). Effect of addition ZrO_2_-Al_2_O_3_ nanoparticles mixture on some properties and denture base adaptation of heat cured acrylic resin denture base material. *Journal of Baghdad College of Dentistry*.

[25] Chen S., Liang W. (2004). Effects of fillers on fiber reinforced acrylic denture base resins. *Mid-Taiwan Journal of Medicine*.

[26] Muklif O. R., Ismail I. J. (2015). Studying the effect of addition a composite of silanized nano-Al2O3 and plasma treated polypropylene fibers on some physical and mechanical properties of heat cured PMMA denture base material. *Journal of Baghdad College of Dentistry*.

[27] Alhareb A. O., Ahmad Z. A. (2011). Effect of Al_2_O_3_/ZrO_2_ reinforcement on the mechanical properties of PMMA denture base. *Journal of Reinforced Plastics and Composites*.

[28] Zhang X.-Y., Zhang X.-J., Huang Z.-L., Zhu B.-S., Chen R.-R. (2014). Hybrid effects of zirconia nanoparticles with aluminum borate whiskers on mechanical properties of denture base resin PMMA. *Dental Materials Journal*.

[29] Moreno-Maldonado V., Acosta-Torres L. S., Barceló-Santana F. H., Vanegas-Lancón R. D., Plata-Rodríguez M. E., Castaño V. M. (2012). Fiber-reinforced nanopigmented poly(methyl methacrylate) as improved denture base. *Journal of Applied Polymer Science*.

[30] Council on Dental Materials and Devices (1975). Revised American dental association specification no. 12 for denture base polymers. *Journal of the American Dental Association*.

[31] British Standard Institution 2000-ISO 1567 (1999). *Dentistry—Denture Base Polymer*.

[32] Novak B. M. (1993). Hybrid nanocomposite material between inorganic and organic polymer. *Advanced Materials*.

[33] Lin G. M., Xie G. Y., Sui G. X. (2012). Hybrid effect of nanoparticles with carbon fibers on the mechanical and wear properties of polymer composite. *Composites Part B: Engineering*.

[34] Ellakwa A. E., Morsy M. A., El-Sheikh A. M. (2008). Effect of aluminum oxide addition on the flexural strength and thermal diffusivity of heat-polymerized acrylic resin. *Journal of Prosthodontics*.

[35] Zhang X. J., Zhang X. Y., Zhu B. S. (2011). Effect of nano-ZrO_2_ on flexural strength and surface hardness of polymethylmethacrylate. *Shanghai Kou Qiang Yi Xue*.

[36] John J., Gangadharand S. A., Shah I. (2001). Flexural strength of heat-polymerized polymethyl methacrylate denture resin reinforced with glass, aramid, or nylon fibers. *Journal of Prosthetic Dentistry*.

[37] Asar N. V., Albayrak H., Korkmaz T. (2013). Influence of various metal oxides on mechanical and physical properties of heat-cured polymethyl methacrylate denture base resins. *Journal of Advanced Prosthodontics*.

[38] Alhareb A. O., Ahmad Z. A. (2010). Effect of Al_2_O_3_/ZrO_2_ hybrid on the fracture toughness and flexural properties of PMMA denture base. *Advanced Materials Research*.

[39] Hameed H. K., Abdul Rahman H. (2015). The effect of addition nano particle ZrO_2_ on some properties of autoclave processed heat cure acrylic denture base material. *Journal of Baghdad College of Dentistry*.

